# Sustained Idiopathic Outflow Tract Ventricular Tachycardia: Clinical Evidence for RVOT Predominance

**DOI:** 10.1111/jce.70315

**Published:** 2026-03-13

**Authors:** Moneeb Khalaph, Nadica Trajkovska, Maxim Didenko, Mustapha El Hamriti, Guram Imnadze, Denise Guckel, Philipp Lucas, Martin Braun, Thomas Fink, Vanessa Sciacca, Ersan Akkaya, Sebastian E. Beyer, Hassan El‐Shirbiny, Maximilian Mörsdorf, Christian Sohns, Philipp Sommer, Angeliki Darma

**Affiliations:** ^1^ Department of Electrophysiology, Heart and Diabetes Center NRW Ruhr University Bochum Bad Oeynhausen Germany; ^2^ Clinic for Cardiology/Electrophysiology GZO‐Spital Wetzikon, Zürich Switzerland; ^3^ Clinic for Electrophysiology University Hospital Ruppin‐Brandenburg Germany; ^4^ Telemachus and Irene Demoulas Family Foundation Center for Cardiac Arrhythmias Massachusetts General Hospital Boston Massachusetts USA; ^5^ Tulane Research Innovation for Arrhythmia Discoveries (TRIAD) Tulane University, School of Medicine New Orleans Louisiana USA

**Keywords:** catheter ablation, LVOT, OT‐VT, outflow tract arrhythmia, premature ventricular contraction (PVC), RVOT, ventricular tachycardia

## Abstract

**Background:**

Idiopathic outflow tract premature ventricular contractions (PVCs) are most commonly localized in the right ventricular outflow tract (RVOT). The clinical significance of sustained outflow tract ventricular tachycardia (OT‐VT) regarding any predominance to one specific anatomic area remains uncertain.

**Subjective:**

This study aimed to characterize the clinical and procedural features of patients with idiopathic sustained OT‐VT and determine whether the site of origin can be predicted from clinical presentation.

**Methods:**

This retrospective cohort included 35 consecutive patients with documented sustained OT‐VT (≥ 30 s), from over 400 patients referred for idiopathic outflow tract arrhythmia ablation. Baseline clinical and echocardiographic characteristics were collected and procedural data reviewed. Sites of arrhythmia origin were identified by electroanatomic mapping and ablation outcomes.

**Results:**

Sustained OT‐VT arose exclusively from right‐sided outflow tract foci, most commonly the postero‐ or anteroseptal RVOT (*n* = 26, 74.3% and *n* = 6, 17.1%, respectively) and less frequently from the posterolateral (*n* = 3, 8.6%). No cases of sustained VT from the LVOT were observed. Acute ablation success was achieved in all patients. During 12 months of follow‐up, recurrence occurred in three patients (8.6%), all of whom were successfully managed with repeat ablation. No complications were observed.

**Conclusion:**

In patients with structurally normal hearts, sustained OT‐VT in our cohort arose exclusively from the RVOT. Recognition of this pattern can guide mapping and ablation strategies.

## Graphical abstract

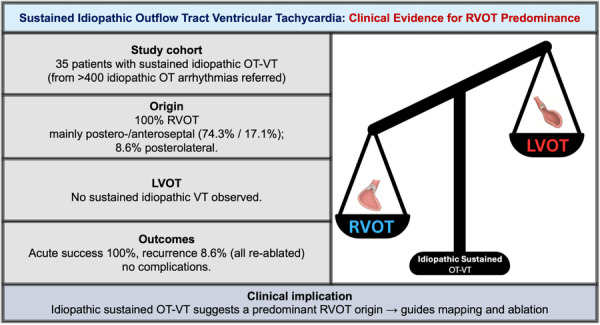

**Abbreviations**
AMC: aortomitral continuity
LVEDD: left ventricular end‐diastolic diameter
LVEDV: left ventricular end‐diastolic volume
LVOT: left ventricular outflow tract
LV‐EF: left ventricular ejection fraction
PVC: premature ventricular contraction
RVOT: right ventricular outflow tract
VT: ventricular tachycardia

## 1 Introduction

Outflow tract (OT) ventricular arrhythmias represent the most common form of ventricular arrhythmia in patients without structural heart disease, accounting for approximately 10% of cases [1]. Their incidence in the general population is rising and is currently estimated at 51.9 per 100 000 adults [2].

The typical clinical manifestation is a high burden of premature ventricular contractions (PVCs); however, up to one third of patients present with non‐sustained or sustained ventricular tachycardia (VT) [2]. These arrhythmias are often highly symptomatic—manifesting as palpitations, dizziness, presyncope, syncope—and, in individuals with a high arrhythmic burden, may progress to tachycardia‐induced cardiomyopathy (2; 7.2 per 100 000 adults).

The objective of this study was to examine the relationship between the clinical presentation of sustained idiopathic outflow tract ventricular tachycardia (OT‐VT) and the precise anatomical site of origin.

## 2 Methods

### 2.1 Study Design and Population

We retrospectively analyzed 35 consecutive patients, enrolled between 2018 and 2025, out of more than 400 patients referred for outflow tract arrhythmia ablation who had documented sustained OT‐VT (≥ 30 s).

Patients with structural heart disease or ventricular tachycardia of non–outflow tract origin were excluded. Structural heart disease was ruled out based on clinical history, multimodality imaging (echocardiography, cardiac magnetic resonance imaging), and, when indicated, coronary angiography. Baseline data were collected immediately prior to the ablation procedure. Sustained ventricular tachycardia was defined according to the 2022 ESC Guidelines for the management of ventricular arrhythmias and the prevention of sudden cardiac death [3]. The study was conducted in accordance with the principles of the Declaration of Helsinki and was approved by the local ethics committee. Furthermore, this manuscript adheres to the anatomic nomenclature recommended by Anderson, Sternick, et al. for describing the location of substrates associated with abnormal cardiac rhythm [4].

### 2.2 Electrophysiological Study and Ablation Strategy

Before the procedure, all patients gave written consent for catheter ablation. The procedures were performed either under conscious sedation using propofol and/or fentanyl or without sedation, depending on patient preference and tolerance. Intravenous heparin was administered to maintain an activated clotting time ≥ 300 s when left ventricular outflow tract (LVOT) or basal LV mapping was performed; otherwise, a prophylactic heparin bolus was given at the start of the procedure. Vascular access was obtained via the femoral veins: two 7F sheaths in the left femoral vein and an 8.5F—with or without steerable sheath (Carto Vizigo, J&J MedTec, Irvine, USA, or Agilis, Abbott, Chicago, USA)—in the right femoral vein. A decapolar catheter (Inquiry, Abbott, Chicago, USA) was advanced into the distal coronary sinus, and a quadripolar catheter (Supreme, Abbott, Chicago, USA) was positioned at the right ventricular apex (RVa).

Right ventricular mapping was performed via the right femoral vein. LV mapping was performed using either a retrograde aortic approach alone or in combination with an antegrade transseptal approach. Transseptal puncture was performed in the anterior‐inferior fossa ovalis under fluoroscopic guidance.

**Figure 1:**
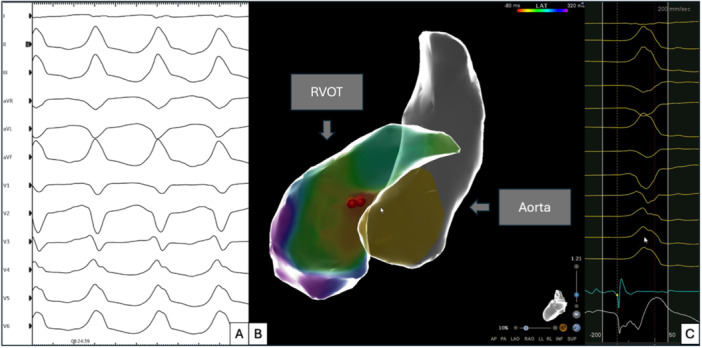
Representative case of a female patient with sustained VT arising from the posteroseptal RVOT. (A) 12‐lead ECG of the clinical VT. (B) Activation mapping of the RVOT and LVOT in a modified posterior–anterior view, showing the earliest activation at the posteroseptal RVOT. (C) 12‐lead ECG of the VT with annotation of the corresponding signal in the CARTO V8 system.

Electroanatomical mapping of the RVOT/LVOT (Carto 3, J&J MedTec, Irvine, USA, or Ensite Precision/X, Abbott, Chicago, USA) was carried out using a bipolar mapping catheter (ThermoCool/SMARTTOUCH SF/QDOT, J&J MedTec, Irvine, USA or FlexAbility/TactiCath/TactiFlex, Abbott, Chicago, USA) to assess voltage and anatomy (Figure 1). Local bipolar voltage < 1.5 mV was defined as low‐voltage, indicating potential arrhythmogenic substrate.

The procedure was initiated with activation mapping of the clinical PVC, when spontaneous PVCs were present, with or without isoprenaline, to identify the site of earliest excitation. Before ablation, programmed ventricular stimulation using a standard drive‐train protocol (S1: 500, 430, 370, 330 ms) [5] was performed to induce the clinical VT and confirm that the PVC and VT shared the same origin. If no spontaneous PVCs were present, VT induction was attempted at the start of the procedure; activation mapping was performed if the VT was hemodynamically tolerated, whereas pace mapping was used in cases of hemodynamic intolerance. Radiofrequency ablation was delivered with a maximum power of 50 W for approximately 60 s per lesion.

The primary endpoint was elimination of the clinical PVC and non‐inducibility of any VT during a 30‐min waiting period. To confirm this, programmed ventricular stimulation and isoprenaline challenge were repeated at the end of the procedure using the same protocol.

### 2.3 Statistical Analysis

Data were recorded in Microsoft Office Excel, Version 16.92 (Microsoft, Redmond, WA, USA) (2024). Statistical analysis was performed using SPSS (IBM SPSS Statistics 28). The level of statistical significance was set at *p* ≤ 0.05. Inferential statistics are intended to be exploratory, not confirmatory, and were interpreted accordingly. The normality of the data distribution was tested using the Kolmogorov–Smirnov test, and the Mann–Whitney *U* Test was used to compare baseline data.

## 3 Results

### 3.1 Patient and Procedural Characteristics

This study included a total of 35 patients who were referred for catheter ablation after experiencing sustained OT‐VT. The mean age was 52.8 ± 11.9 years, and 71% were male. The mean left ventricular ejection fraction was 56.4% ± 9.6%, and 90.1% of patients were receiving β‐blocker therapy at baseline. Detailed baseline characteristics are presented in Table 1.

**Table 1:** Baseline characteristics.

Variable	OT‐VT Group (*n* = 34)	PVC Group	*p* value
Age (years)	52.8 ± 11.9	60.7 ± 15.7	< 0.05
Male gender (%)	71	57	< 0.05
Art. Hypertension (%)	32.6	35.4	0.835
Diabetes mellitus (5%)	11.5	13.6	0.785
LV‐EF (mean %, ±SD)	56.4 ± 9.6	57.2 ± 7.9	0.831
Cardiac MRI (%)	84.8	21.4	< 0.05
BMI (mean, ±SD)	24.9 ± 4.2	26.2 ± 5.3	0.657
Betablocker (%)	90.1	88.3	0.791
Antiarrhythmic drug (%)	27.3	11.6	< 0.05

The median procedure duration was 54 min [IQR 49–60], with a median fluoroscopy time of 1.9 min [IQR 1.3–2.7] and a radiation dose of 105 cGy·cm² [92–116]. Procedural characteristics are summarized in Table 2.

**Table 2:** Procedural and ablation parameter.

Variable	Total (*n* = 35)
Spontaneous/induced PVC	27 (77%)
VT induced	22 (63%)
Procedure time (min, [IQR]))	54 [49–60]
Fluoroscopy duration (min, [IQR])	1.9 [1.3–2.7]
Fluoroscopy dose (cGy)*cm^2^), [IQR]	105 [92–116]

Site of origin	
RVOT	35 (100%)
Posterolateral RVOT	3 (8.6%)
Posterolateral RVOT	26 (74.3%)
Anteroseptal RVOT	6 (17.1%)
Parahisian	0 (0%)
RVOT free wall	0 (0%)

LVOT	0 (0%)
LVOT‐Cusps	0 (0%)
AMC/Basal LV	0 (0%)
LV‐Summit	0 (0%)
Acute success	35 (100%)
Complications	0 (0%)
Recurrence	3 (8.6%)

### 3.2 VT Inducibility and Site of Origin

**Figure 2:**
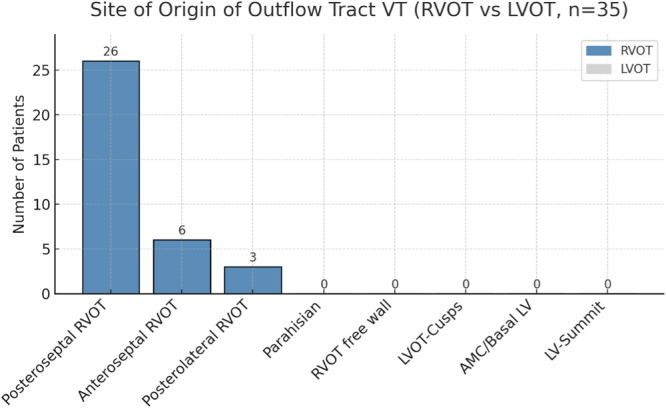
Distribution of successful ablation sites in patients with OT‐VT. All sites of origin were localized within the RVOT, predominantly in the posteroseptal region, followed by the anteroseptal and posterolateral RVOT. No arrhythmias originated from the LVOT. (AMC, aortomitral continuity; LV, left ventricle; LVOT, left ventricular outflow tract; RVOT, right ventricular outflow tract).

Spontaneous PVCs with the same morphology as the clinical VT were documented in 27 patients (77%), and their elimination was used as a procedural endpoint. Sustained clinical VT was inducible in 22 patients (63%). All 35 patients underwent successful ablation, with non‐inducibility of the clinical VT or PVC confirmed at the end of the procedure. The predominant anatomical focus was the posteroseptal RVOT (*n* = 26, 74.3%), followed by the anteroseptal RVOT (*n* = 6, 17.1%) and the posterolateral RVOT (*n* = 3, 8.6%). Neither the RVOT free wall nor the parahisian region was identified as a site of origin in this cohort. Notably, none of the patients with sustained OT‐VT had a left‐sided arrhythmia focus (LVOT, coronary cusps, aortomitral continuity, or basal LV, Figure 2).

### 3.3 Complications and Follow‐Up

**Figure 3:**
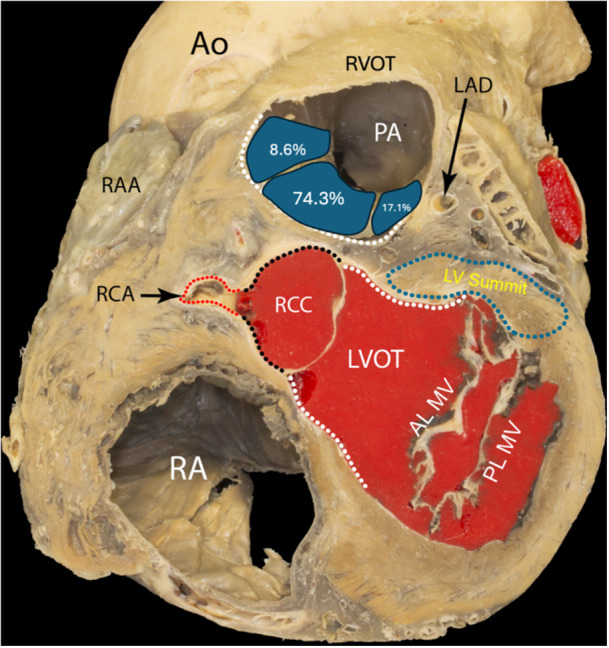
Schematic illustration of ventricular arrhythmia origins in our cohort, with successful ablation sites at the posteroseptal RVOT (74.3%), anteroseptal RVOT (17.1%), and posterolateral RVOT (8.6%). The figure highlights the close anatomical relationship between RVOT and LVOT. (Ao, aorta; AL/PL MV, anterior/posterior mitral valve leaflet; LAD, left anterior descending coronary artery; PA, pulmonary artery; RA, right atrium; RAA, right atrial appendage; RCC, right coronary cusp; RCA, right coronary artery).

No complications occurred during or after the procedures. After a follow‐up of 12 months, three patients (8.6%) experienced recurrence of clinical VT and underwent a successful repeat ablation. The remaining 32 patients (91.4%) remained free of ventricular arrhythmias and experienced no major adverse events during the follow‐up period (Figure 3).

## 4 Discussion

This study highlights the following major findings.

− In OT ventricular arrhythmias, the clinical presentation can provide important clues to the site of origin
− In our cohort, sustained OT‐VT arose exclusively from right‐sided foci, most frequently within the postero‐ or anteroseptal RVOT
− Common left‐sided sites of origin for ventricular arrhythmias (LVOT, coronary cusps, or basal LV regions such as the aortomitral continuity or LV summit) were not observed in patients with sustained idiopathic OT‐VT in this cohort
− The presence of sustained left‐sided VTs may instead suggest underlying structural heart disease

## 5 Embryological Insights

Embryological development provides a framework for understanding the predisposition of the RVOT to arrhythmogenesis. The right and left ventricular outflow tracts develop from the incorporation of the truncus arteriosus into the muscular ventricular components during septation. Although they share this common origin, their genetic programs diverge: Tbx5 is expressed in the LVOT but not in the RVOT [6]. Moreover, myocytes in the outflow tract regions acquire fast conduction properties relatively late compared with the working myocardium. The early cells recruited for outflow tract elongation are morphologically primitive with poorly developed sarcomeres, and it has been proposed that the RVOT in particular retains more of these embryonic features. This persistence of an immature phenotype may contribute to the enhanced automaticity frequently observed in the outflow tract regions [6]. Even in the mature heart, the RVOT and LVOT remain closely related, separated only by thin myocardial and fibrous layers. This proximity explains the frequent overlap in ECG morphologies and the need for detailed mapping across both regions [7] (Figure 2).

## 6 Mechanistic Substrate

The RVOT combines structural and functional features that favor arrhythmogenesis. It is a thin‐walled, free‐standing structure with myocardial extensions into the pulmonary artery and dense autonomic innervation [8, 9]. In contrast, the LVOT is characterized by greater fibrous continuity with the aortic and mitral annuli, providing less excitable myocardial tissue and therefore a substrate less favorable for sustaining tachycardia. Electrophysiologically, RVOT tachycardias are mediated by cAMP‐dependent delayed afterdepolarizations, which are catecholamine‐sensitive, inducible with isoproterenol, and suppressed by adenosine [7, 10]. This mechanism is particularly relevant for sustained VT: under adrenergic stimulation, delayed afterdepolarizations can recur repetitively, enabling triggered activity to propagate long enough to maintain tachycardia even in the absence of structural disease. Clinically, this explains why RVOT arrhythmias often occur during stress or exertion, and why β‐blockade or sedation can reduce inducibility [11]. These same properties also account for variability in mapping and ablation outcomes depending on intraprocedural autonomic tone [12].

## 7 Right‐Sided Predominance

Against this embryological and mechanistic background, it is not surprising that in our cohort sustained OT‐VT arose exclusively from right‐sided foci, most commonly the postero‐ or anteroseptal RVOT. This finding is consistent with earlier reports that identify the RVOT as the dominant site of idiopathic ventricular arrhythmias in structurally normal hearts [7]. The absence of left‐sided origins reinforces the concept that intrinsic developmental and anatomical features endow the RVOT with a greater arrhythmogenic potential. Clinically, this finding highlight that sustained OT‐VT in a structurally normal heart could suggest a right‐sided source.

## 8 Sustained LVOT VT: Truly Idiopathic or Substrate‐Related?

In contrast to the RVOT, sustained monomorphic VT originating from the LVOT appears to be uncommon in structurally normal hearts and has frequently been associated with an underlying substrate. Several studies have demonstrated that LVOT or periaortic tachycardias initially classified as “idiopathic” often localize to small regions of myocardial scar, particularly around the basal septum or periaortic region [13, 14]. High‐resolution mapping in nonischemic cardiomyopathy has further shown that the LVOT and adjacent periaortic regions may serve as sources of reentrant VTs, with many fulfilling criteria for scar‐related reentry rather than triggered activity [15]. Likewise, basal‐septal VTs mapped near the LVOT have been described in the context of structural disease, where intramural substrate rather than focal automaticity accounted for the arrhythmia mechanism [16].

Iwai et al. reported that more than one‐third of LVOT arrhythmias in their cohort presented as sustained VT, with electrophysiological properties similar to those of RVOT arrhythmias [17]. Other reports have described sustained arrhythmias originating from the aortomitral continuity [18] or periaortic regions, where detailed mapping has often revealed previously unrecognized substrate [15]. More recently, larger ablation series have highlighted intramural LVOT arrhythmias as a distinct clinical entity [19]. The absence of sustained LVOT VT in our cohort is therefore likely related to the relatively uncommon clinical presentation of sustained OT‐VT in structurally normal hearts—despite a large number of patients undergoing PVC ablation from the same referral base—as well as the possibility that some LVOT foci, particularly intramural or periaortic origins, may reflect subtle or occult structural disease that would not have met our inclusion criteria. It is therefore possible that at least a proportion of LVOT cases described in prior studies may not have been truly idiopathic, but rather early manifestations of structural heart disease.

## 9 Clinical Implications

Together, these insights emphasize that the clinical manifestation of sustained OT‐VT in structurally normal hearts is a marker of a right‐sided, RVOT origin. The convergence of embryological predisposition, anatomical and electrophysiological vulnerability, and adrenergic sensitivity provides a coherent explanation for why sustained tachycardia was confined to the RVOT in our cohort. Recognizing this pattern enables more efficient mapping, reduces procedure time, and improves ablation success. A deeper understanding of the developmental background and autonomic modulation of outflow tract arrhythmias strengthens both diagnostic accuracy and therapeutic outcomes.

## 10 Limitations

This study has several limitations. First, it represents a retrospective, non‐randomized analysis from a single tertiary referral center, introducing the possibility of referral and selection bias. Second, the sample size was relatively small, which limits statistical power and the generalizability of the findings. Third, the follow‐up period was of limited duration, and longer‐term recurrence rates may therefore be underestimated. Finally, the programmed ventricular stimulation protocol applied was designed primarily for scar‐related VT and may not have been optimal for induction of idiopathic outflow tract arrhythmias.

## 11 Conclusion

In patients with outflow tract ventricular arrhythmias, the presence of sustained VT suggests an RVOT origin—most commonly postero‐ or anteroseptal—and may serve as a practical clinical indicator to guide mapping and ablation strategies, highlighting the limited contribution of the LVOT in idiopathic sustained outflow tract arrhythmias.

## Funding

The authors received no specific funding for this work.

## Conflicts of Interest

C.S. received research support and lecture fees from Medtronic, Abbott, Boston Scientific, and J&J MedTec; he is a consultant for Medtronic, Boston Scientific, and J&J MedTec. P.S. is Member of Advisory Board for Abbott, Boston Scientific, J&J MedTech and Medtronic. The other authors declare no conflicts of interest.

## Data Availability

The data that support the findings of this study are available from the corresponding author upon reasonable request.
